# Artificial Intelligence and Deep Learning in Sensors and Applications: 2nd Edition

**DOI:** 10.3390/s25041144

**Published:** 2025-02-13

**Authors:** Yu-Heng Hsieh, Shyan-Ming Yuan

**Affiliations:** Department of Computer Science, National Yang Ming Chiao Tung University, Hsinchu 30010, Taiwan; k28998989.cs11@nycu.edu.tw

## 1. Introduction

In the era of rapidly advancing technologies, the integration of artificial intelligence (AI) and deep learning (DL) with sensor technologies is providing novel solutions across diverse fields, from healthcare [1,2,3,4,5] to industrial applications [6,7,8,9,10]. These innovations enable more efficient data analysis, accurate predictions, and improved performance in real-world scenarios. This Special Issue brings together a collection of research articles that showcase the impact of AI and DL on sensor applications, highlighting their ability to address complex challenges such as sensor fault detection, environmental adaptations, and predictive maintenance.

The contributions to this Special Issue illustrate the transformative potential of AI in various domains. For example, AI-driven systems have enhanced the classification of animal vocalizations, traffic flow prediction, and fault diagnosis in critical infrastructure systems such as power grids. Moreover, the use of AI to improve the performance of resource-constrained embedded systems demonstrates its capacity to adapt to dynamic environments and optimize system efficiency. Furthermore, innovations in additive manufacturing and medical imaging underscore the broad applicability of AI techniques in solving complex problems with high accuracy.

Despite the promising outcomes, this Special Issue also emphasizes the challenges that remain in the field, such as data scarcity, model robustness, and the need for improved real-world implementation. The papers highlight the importance of refining algorithms, enhancing the generalization capabilities of deep learning models, and developing more efficient sensor systems. As AI and deep learning advance, their integration with sensor technologies is poised to play a pivotal role in shaping the future of intelligent systems across diverse applications. Future research will focus on overcoming these challenges and further advancing the capabilities of AI-driven sensor technologies to solve increasingly complex real-world problems.

## 2. Overview of Published Papers

Submissions were rigorously evaluated for their technical quality, leading to the selection of ten research articles and two review papers for inclusion in this Special Issue. These works focus on advancements in weed detection through deep learning and the application of explainable artificial intelligence (XAI) in medical contexts. The selected contributions are listed below, along with concise summaries of each.

In Contribution 1, the authors introduce a streamlined framework designed to detect and classify wildlife sounds, offering a solution to the inefficiency of manual audio analysis. By segmenting relevant sound regions and using STFT, MFCCs, and LFCCs for feature extraction, the system achieves high classification accuracy with CNN models like AlexNet and ResNet. It offers a robust tool for behavior analysis and highlights the potential of deep learning in bioacoustics, with room for further improvements.

In Contribution 2, the authors introduce the Dynamic Spatio-Temporal Memory-Augmented Network (DSTMAN), designed to address challenges in traffic forecasting, such as effectively capturing spatial and temporal patterns and dealing with complex temporal behaviors. DSTMAN employs multiple spatial and temporal representations to adapt to changing environments, integrates them into a scalable framework for extracting hierarchical dependencies, and incorporates a memory mechanism to adaptively construct a neighborhood graph. The model demonstrates superior performance, achieving a 4% reduction in MAE compared to MTGNN, a 6.9% reduction compared to DCRNN, and a 5.8% reduction compared to AGCRN, highlighting its effectiveness in analyzing spatial and temporal data.

In Contribution 3, this study addresses the resilience of AI systems in resource-constrained, safety-critical embedded systems by introducing a system design and training approach that incorporates adaptive neural networks. The approach enhances the robustness of convolutional networks by 24% and visual transformers by 19.7% under fault injections, while achieving a 16.9% and 21.6% increase in resilience for ResNet-110 and DeiT-S, respectively, under adversarial attacks. Additionally, it reduces computational resource consumption by over 30%. Meta-training further improves task resilience by an average of 22%, demonstrating a cost-effective solution for resilient AI in resource-limited environments.

In Contribution 4, the authors introduce the RF-IWOA-GRU temperature compensation model to address accuracy challenges in sensors affected by temperature fluctuations. The model uses Random Forest (RF) to estimate missing data in time-series records and combines Gated Recurrent Unit (GRU) networks with an Improved Whale Optimization Algorithm (IWOA) to correct for heat-induced discrepancies. The system takes advantage of GRU’s ability to store and process past information and IWOA’s superior optimization performance to improve the accuracy and consistency of sensors. Testing outcomes show that the RF-IWOA-GRU system surpasses traditional methods, reducing the standard deviation of sensors from 10.18 kPa to 1.14 kPa. Additionally, the mean absolute error is reduced by 75.10%, while the root mean squared error is decreased by 76.15%, compared to conventional approaches.

In Contribution 5, the authors introduce the BNN-Clip training scheme to enhance the efficiency of binary neural networks (BNNs) by increasing parallelism and improving data flow. The method incorporates a compression module that lowers the data bit-depth from 32 bits to 8 bits and reduces the processing capacity of binary layers, maintaining accuracy without compromise. Additionally, the study optimizes the batch normalization layer to minimize delay and streamline deployment. The study also presents an improved approach to binary convolution operations designed specifically for ARM NEON instruction sets. The experiments reveal substantial reductions in inference delay, with speed-ups reaching 1.3× and 2.4× compared to two advanced BNN frameworks, all while preserving accuracy levels comparable to the best models on benchmarks like CIFAR-10, SVHN, and ImageNet.

In Contribution 6, this article introduces a novel method for enhancing the wire-arc additive manufacturing (WAAM) process for grid-like frameworks by integrating pool stress analysis with image recognition algorithms. The study aims to improve the precision of molten pool state predictions, tackling challenges such as fractured rods and surface morphology imperfections. The optimized U-net model for molten pool segmentation achieved high accuracy, with 98.18% accuracy, 96.64% MIOU, and 98.34% recall. Additionally, a method for estimating the equilibrium of forces in the molten pool and predicting its states (normal, sagging, and collapsing) is proposed. The experimental results show a 90% prediction accuracy for the testing set, enabling effective monitoring and preemptive avoidance of issues during printing. This approach aids in optimizing the process parameters and enhancing welding quality, paving the way for intelligent, unmanned WAAM and AI-driven monitoring systems in manufacturing.

In Contribution 7, the authors present an innovative approach to diagnosing insulator faults by combining Rocket algorithms with empirical mode decomposition (EMD) methods and machine learning classifiers, such as CEEMDAN, EWT, and VMD. By utilizing advanced signal processing techniques to capture key information from time series data, the integration of EMD methods with MiniRocket greatly improves fault classification performance using logistic regression. The results demonstrate exceptional classification rates, achieving 0.992 with CEEMDAN, 0.995 with EWT, and 0.980 with VMD. These findings emphasize the value of merging EMD techniques with cutting-edge machine learning approaches to enhance the resilience and reliability of power distribution networks.

In Contribution 8, this study presents a novel method for creating a comprehensive dataset designed to tackle inverse challenges in electromagnetic diagnostic imaging. The dataset consists of 25,000 distinct objects generated by randomly combining ellipses and polygons with varying electrical properties, enabling deep learning models to precisely reconstruct permittivity profiles in complex imaging scenarios. By introducing variability into the data, the dataset enhances the training of a U-net model, improving its flexibility and minimizing the potential for overfitting. Evaluating the trained model on three separate datasets yielded impressive results, with resemblance indices greater than 0.84, normalized root mean square errors under 14%, and peak signal-to-noise ratios surpassing 33 dB. These findings highlight the effectiveness of this dataset in training deep learning models to address inverse challenges in diagnostic imaging, eliminating reliance on additional computational methods.

In Contribution 9, this paper investigates the use of generative adversarial networks (GANs) to generate realistic 3D magnetohydrodynamic (MHD) distortion simulations, simulating distortions similar to those observed in magnetic resonance imaging (MRI), and applies them to enhance existing electrocardiogram (ECG) datasets. The augmented data was evaluated using similarity metrics and the performance of a deep learning (DL)-based R-peak detector. The results demonstrated that the GAN-generated MHD distortions closely resembled real distortions and significantly improved the R-peak detector’s accuracy and recall when trained with augmented ECG data. This approach effectively addresses the limitations of sparse or biased ECG datasets by using synthetic data, enhancing the generalization capacity of DL models for ECG analysis without needing to collect additional patient data.

In Contribution 10, this paper reviews machine learning approaches aimed at personalizing auditory device amplification settings. It first highlights the limitations of the current generic models for auditory device prescriptions. The review then discusses various research efforts in engineering and audiology that focus on adjusting amplification settings to suit individual user preferences and specific audio environments. The paper compiles a comprehensive collection of research on personalizing auditory device adjustments and underscores the ability of machine learning to improve the hearing experience for users. It wraps up by discussing the obstacles and proposing directions for further investigation in this field.

## 3. Conclusions

This Special Issue showcases the transformative potential of artificial intelligence (AI) and deep learning in conjunction with sensor technologies, offering innovative solutions across a wide array of fields, from medical diagnostics to industrial applications. The diverse approaches presented in these papers emphasize the importance of personalized and efficient solutions in domains such as healthcare, resource-constrained embedded systems, traffic flow prediction, and additive manufacturing. The integration of machine learning models, particularly deep learning, has proven to be a powerful tool in enhancing sensor data analysis, improving accuracy, and optimizing performance.

In particular, the papers highlight the use of AI to address complex real-world challenges, such as sensor fault detection, dynamic environmental adaptations, and predictive maintenance. The role of data augmentation, neural networks, and hybrid algorithms is emphasized as key components in achieving high accuracy and generalizability in various domains. Furthermore, the advances in handling spatio-temporal data and optimizing resource use in embedded systems demonstrate the broad applicability of AI-driven techniques.

While the results presented are promising, this Special Issue also underscores the need for further research to overcome challenges such as data scarcity, model robustness, and the integration of AI in real-world industrial settings. Ongoing efforts should focus on refining algorithms, optimizing sensor systems, and expanding the generalization capabilities of deep learning models. Ultimately, this collection of papers illustrates the ongoing evolution of AI and deep learning technologies and their potential to revolutionize how we extract, process, and analyze sensor data in solving real-world problems.
